# Supplementary figures and images for: A conserved Hsp70 phosphorylation regulates cell cycle progression after DNA damage
Source: bioRxiv. 2025 Sep 6:2025.09.05.672953. Preprint. [Version 1] doi: 10.1101/2025.09.05.672953 (PMC12424849; doi:10.1101/2025.09.05.672953)

a)

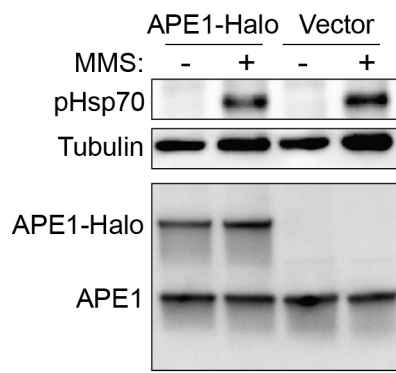

b)

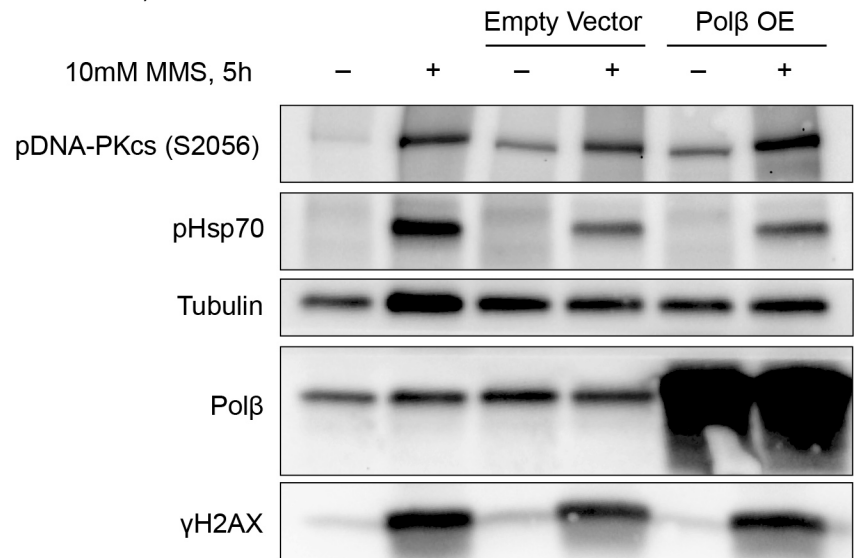

c)

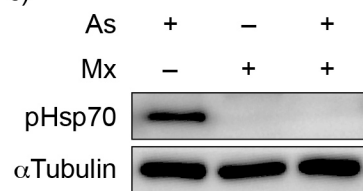

d)

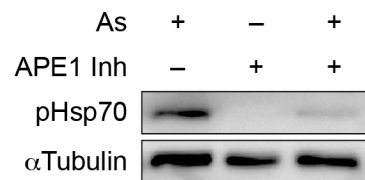

Supplement: Supplement 1 [file media-1.pdf]

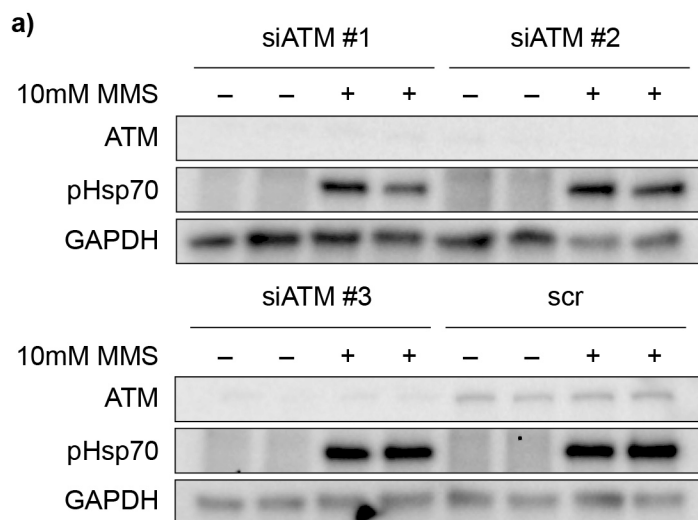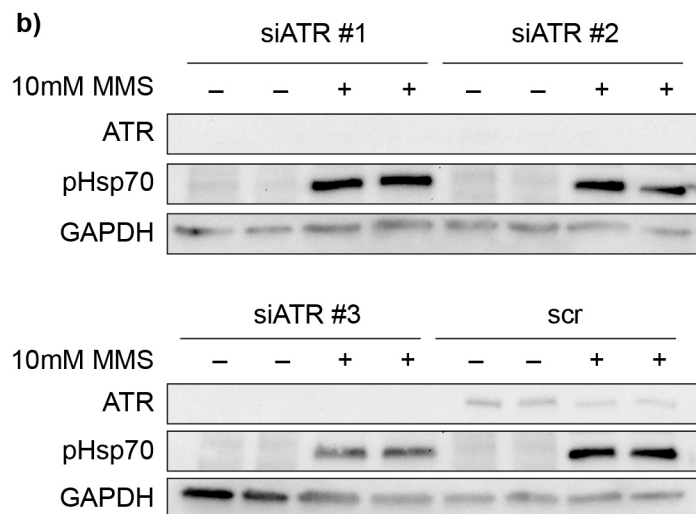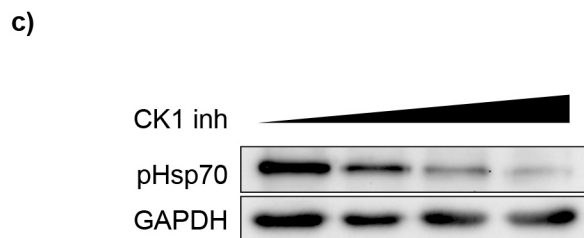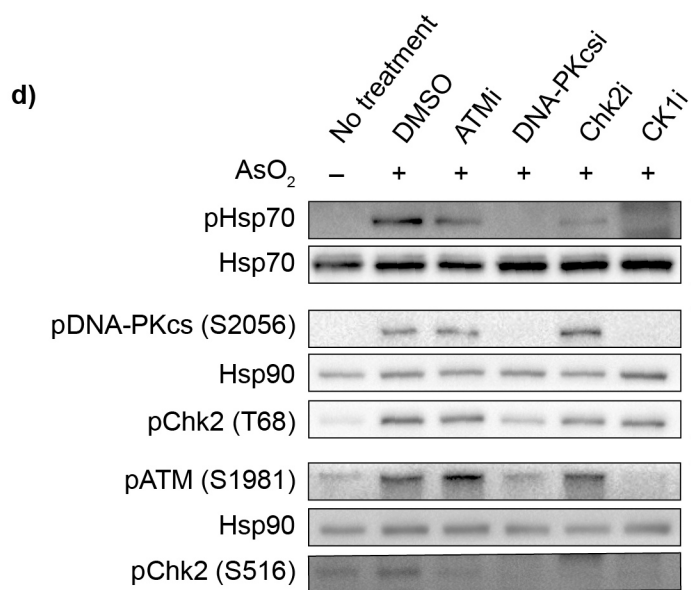

Supplement: Supplement 2 [file media-2.pdf]

a)

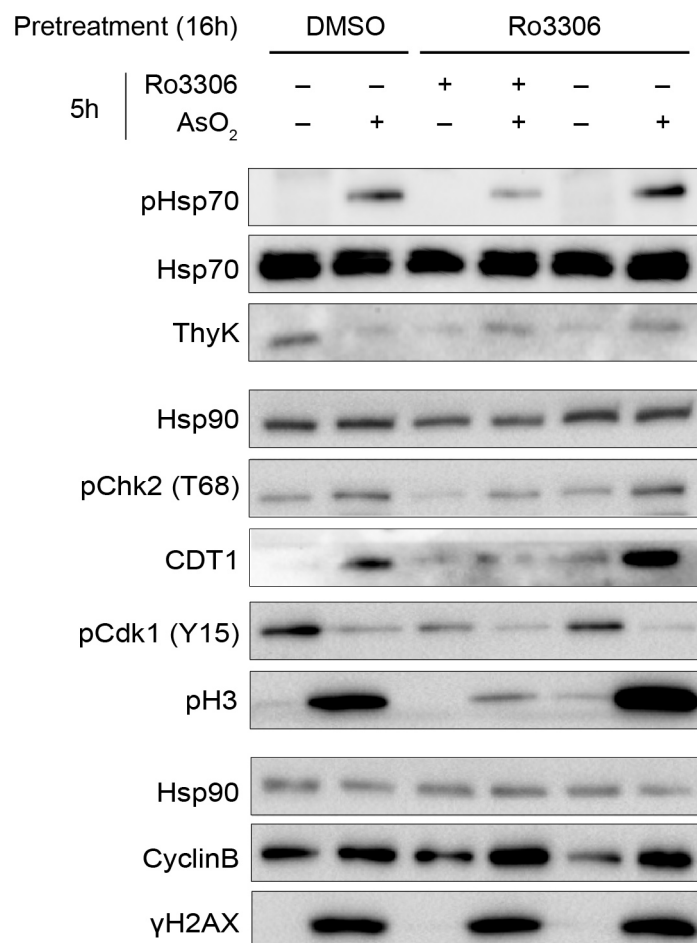

Supplement: Supplement 3 [file media-3.pdf]

a)

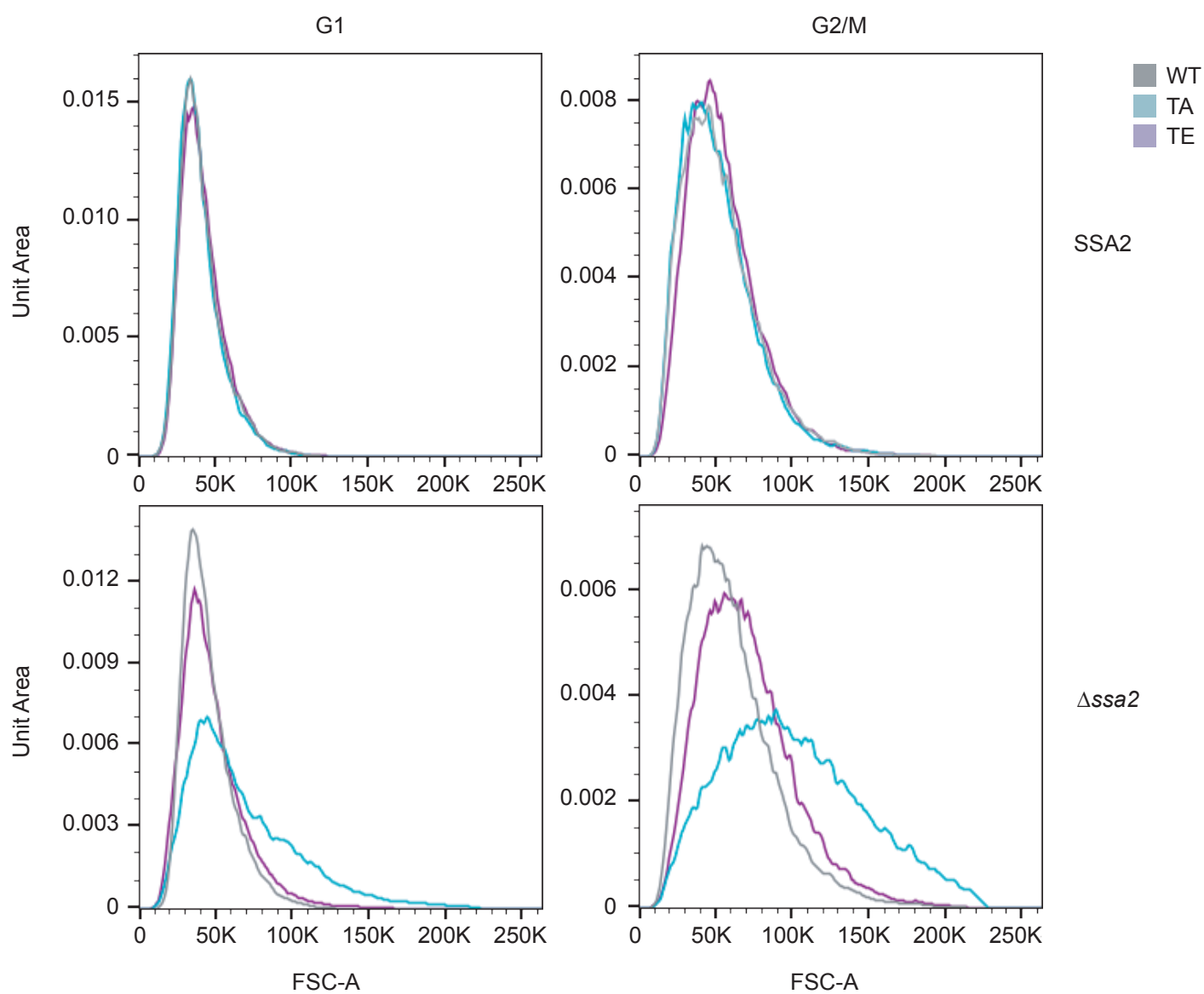

Supplement: Supplement 4 [file media-4.pdf]
